# Treatment outcomes and associated factors among chronic ambulatory heart failure patients at Jimma Medical Center, South West Ethiopia: prospective observational study

**DOI:** 10.1186/s12872-023-03055-8

**Published:** 2023-01-17

**Authors:** Erkihun Assefa, Elsah Tegene, Abinet Abebe, Tsegaye Melaku

**Affiliations:** 1 School of Pharmacy, College of Medicine, and Health Sciences, Mizan-Tepi University, Mizan-Teferi, Ethiopia; 2 Department of Internal Medicine, Jimma Medical Center, Jimma, Ethiopia; 3 School of Pharmacy, Institute of Health, Jimma University, Jimma, Ethiopia

**Keywords:** Heart failure, Treatment outcomes, Associated factors, Ethiopia

## Abstract

**Background:**

Heart failure has been one of the major causes of hospitalization across the world. Focusing on the treatment outcomes of ambulatory heart failure patients will reduce the burden of heart failure such as hospitalization and improve patient quality of life. Even if research is conducted on acute heart failure patients, there is limited data about treatment outcomes of chronic ambulatory heart failure patients. Therefore, this study aimed to assess treatment outcomes and associated factors of chronic ambulatory heart failure patients at Jimma Medical Center, South West Ethiopia.

**Methods:**

A hospital-based prospective observational study was conducted on 242 chronic ambulatory heart failure patients at Jimma Medical Center from November 2020 to June 2021. The data were collected with pretested data collection format, and analyzed with Statistical Package for Social Sciences version 23. Both univariate and multivariate logistic regression model were used to identify factors associated with treatment outcomes of outpatient heart failure, and with a reported *p* value < 0.05, 95% confidence interval (CI) was considered statistical significance.

**Result:**

From 242 patients, 126 (52.1%) were males and 121 (50.0%) patients were aged between 45 and 65 years. Regarding treatment outcomes, 51 (21.1%) of patients were hospitalized, and 58 (24.0%) and 28 (11.6%) of patients had worsened and improved clinical states respectively. Clinical inertia [AOR = 2.820; 95% CI (1.301, 6.110), *p* = 0.009], out-of-pocket payment [AOR = 2.790; 95% CI (1.261, 6.172), *p* = 0.011] and New York Heart Association class II [AOR = 2.534; 95% CI (1.170, 5.488), *p* = 0.018] were independent predictors of hospitalization.

**Conclusion:**

Hospitalization of ambulatory heart failure patients was relatively high. More than half of the patients had clinical inertia. And also, this study showed most ambulatory HF patients had inadequate self-care. Clinical inertia, out-of-pocket payment, and New York Heart Association class II were independent predictors of hospitalization in ambulatory heart failure patients. Therefore, it is better to give more attention to ambulatory heart failure patients to prevent hospitalization and the burden of heart failure.

## Introduction

Heart failure (HF) is a cardiac problem which is a complex clinical syndrome resulting from any structural or functional impairment of ventricular filling or ejection of blood. The resulting decrease in cardiac output is mediated by the body’s neurohormonal response. Ultimately, the permanent changes in the heart muscle structure lead to myocardial remodeling and hypertrophy [1–3]. The clinical presentation of HF can be shortness of breath, decreased exercise tolerance, fatigue, fluid retention, crackles in the lung, and the like [4, 5].

People living with HF require integrated multidisciplinary care and support patient therapeutic education and regular consultations to monitor symptoms, adjust medication, and assess the need for cardiac devices [6]. Management of HF includes both lifestyle modification and pharmacological, majority of patients regardless of symptom severity, require lifelong optimal medical treatment which includes angiotensin-converting enzyme inhibitors (ACEIs), beta-blocker (BB), and mineralocorticoid receptor antagonists (MRA) that decrease hospitalization and mortality [2, 7, 8]. Effective multidisciplinary services for people with chronic heart failure (CHF) can have a positive effect on patient’s life expectancy and quality of life, and it can help to reduce recurrent hospital stays by 30–50% [9].

Despite advances in therapies and prevention, morbidity is still high and quality of life remained poor. It is one challenging public health problems of the twenty-first century and is associated with poor outcomes, such as death and hospitalization. HF admission rate (> 50%) with a mortality rate between 10 and 15%, and hospitalization rate within 6 months after discharge is 30–40% [10]. As the studies showed in Europe, the HF readmission rates were between 26.4% and 51.3% [11, 12]. According to a comprehensive review, HF was found to be associated with a hospital case-fatality rate of 9–12.5% in Sub-Sahara Africa (SSA)increased length of hospitalizations, and a high rate of patient readmission (34.9%) [4]. There are different factors that affect the outcome of ambulatory heart failure patients. Chronic kidney disease, ischemic heart disease, diabetes mellitus, and chronic obstructive pulmonary disease, older age, reduced ejection fraction, higher New York Heart Association (NYHA) class, history of HF hospitalization, lower body mass index and lower diastolic blood pressure, increased risk hospitalization [13, 14]. Ambulatory HF clinic play a major role in avoiding hospitalization, unscheduled visits, emergency intervention, and monitoring and follow-up of the ongoing status of HF patients [15]. As far as our knowledge, available evidence in the literature focus on treatment outcome in inpatient heart failure patients. There is a few studies conducted on treatment outcomes of ambulatory heart failure patients in developed countries. In addition, this study assessed the clinical inertia and self-care behavior of the patient which was not assessed in the available studies. There is no studies describing the health status and treatment outcomes across outpatient heart failure in Ethiopia. Therefore, this study aimed to assess the treatment outcome of ambulatory heart failure patients and its associated factors. This study also provides as evidence to assessed patient’s health status, self-care practice, treatment and which helps to prevent hospitalization and improve patient treatment outcomes at Jimma Medical Center Sout West Ethiopia.

## Methods and materials

### Study design setting and period

A prospective observational hospital-based study was conducted at Jimma Medical Center (JMC) at chronic follow up ambulatory heart failure patients from November 2020-June to 2021. Jimma medical center located in Oromia Regional State is located 352 km from the capital city of Ethiopia, Addis Ababa. It is the only medical center in South-West Ethiopia. Currently, it is providing services for approximately 15,000impatients, 160,000 outpatient attendants, 11,000 emergency cases, and 4500 deliveries a year coming to the hospital from the catchment population of about 20 million people. The medical services provided include: internal medicine which includes: cardiology inpatient and outpatient units, oncology, surgery, orthopedics, ophthalmology, pediatrics, gynecology and obstetrics, dermatology, psychiatric service, pathology, pharmacy, medical laboratory, intensive care unit, radiology and others as both inpatient and outpatient services.

### Study population and source population

All adult chronic HF patients on follow-up at the ambulatory units of JMC were considered as a source of population. Patients who were willing to participate and who fulfill eligibility criteria were taken as the study population. All chronic HF patients with age ≥ 18 years and who had regularly followed-up a minimum of three months prior to the data collection period at ambulatory unit of Jimma Medical Center were included in the study. Patients who were decomposed HF patients, and un willing to participate were excluded in the study.

### Sample size determination and sampling technique

The sample size was determined by using the single population proportion formula and the proportion was taken as 50% since there is no similar study with this title. So, the proportion was taken as 50%, with considering a 95% confidence interval (CI) and 5% marginal error. By considering 10% contingency, the final minimum sample size was 253**.** From those ambulatory chronic heart failure patients enrolled initially, six patients lost to follow-up within the first three months of the study period. An additional five patients were lost to follow-up in the next months of the study period, and 242 patients were included in the final analysis. A convenient sampling technique was used during recruit period. The sample size was calculated as follows: -

$${\text{n}} = \frac{{\left( {{\text{Z}}\upalpha /2} \right)^{2} {\text{p}}\left( {1 - {\text{p}}} \right)}}{{{\text{d}}^{2} }}$$

where n, Required sample size; Z, Standard deviation normal value at 95% CI which is 1.96; p, Proportion of hospitalization ambulatory HF patients; d, Possible margin of error that can be tolerated 5% (0.05). 1 − p, proportion of population that do not possess the character of interest; ${\text{n = }}\frac{{\left( {{1}.{96}} \right)^{{2}} \left( {0.{5}} \right) \, \left( {0.{5}} \right)}}{{\left( {0.0{5}} \right)^{{2}} }} = 384$.

The source population of cardiac patients is 576 which is < 10,000, a population correction formula is used to determine the adjusted minimum sample size.

$${\text{nf}} = \frac{{\text{n}}}{{{1} + {\text{n}}/{\text{N}}}}$$

where n= Initial sample size (384); N=total number of adult cardiac ambulatory patients (576); nf= minimum final sample size was

Thus $${\text{nf}} = \frac{{{384}}}{1 + 384/576} = 230$$.

By considering 10% contingency, the final minimum sample size was **253**

### Study variables

At ambulatory heart failure patients’ hospitalization was the primary outcome of this study. Which means hospitalization was the dependent variable. Socio-demographic (sex, age, residence, living condition, occupational, marital status, educational status), comorbidity, number of comorbidity, duration of HF, NYHA class, etiology of HF), echo cardiographs), behavioral related (exercise, alcohol, chat, smoking coffee and self-care practice, types of medication use, number of medications used, follows-up interval, ways of getting medication, keep regular appointment were independent variable affecting hospitalization of ambulatory heart failure patients.

### Data collection procedure

Data were collected by using pretested data collection format by three trained B. pharm pharmacists. The data collection tool was prepared from similar literature. The tool has two parts patient interviews and chart review. It includes socio-demographic, behavioral, medication-related, and laboratory as well as clinical and diseases-related character of the patient. Initially, chronic heart failure patients were screened based on eligibility criteria. Informed consent was taken for each patient and two months recruitment period was given to fill the sample. Then the patients were followed for six months. Last three months’ follow-up data were taken as a baseline. And follow up clinical data and the patient’s condition were collected according to patients regular follow-up within the study period. The data were collected both through patient interviews and review of medical chart. With regarding self-care behaviour scale of the patient was calculated based on nine items of European HF self-care behaviour scale. The total self-care score of the patient range, a minimum 9 and maximum 45. Then converted in standard self-care score by using the formula total self-care score* by 2.7777. If a standard score ≥ 70 was defined as adequate, and if < 70 was inadequate self-care practice [16].

### Data quality control and assurance

To ensure the quality of data, pre-test was conducted on 5% of sample size patients and necessary modification were done. Regular supervision was taken for data collectors by principal investigator to check the completeness, clarity and accuracy of data to be conducted. Unclear and incomplete data were excluded from analysis.

### Data entry and analysis

The data were coded, cleaned, entered, and compiled to Epi-data version 4.6, and exported to (SPSS version 23.0) used for all statistical analysis. Descriptive statistics, mean, standard deviation, and range were used for continuous variables, while frequency and percentages for categorical variables were used to summarize the results. Logistic regression (univariate and multivariate) models were used to identify factors were associated with the hospitalization of ambulatory heart failure patients. First univariate logistic regression model was analyzed, variables having *p* value < 0.25 were candidate to multivariate logistic regression, and all variables having *p* vale < 0.25 were run to multivariate logistic regression. Final variables having *p* value < 0.05 dictates statistically significance and results were reported as 95% confidence intervals.

### Outcome measures and validating methods

The primary outcome of this study whether the patient was hospitalization or not and it was measured if the patient was admitted to JMC within the study period by reviewing the patient chart report which signatured by an attendant physician. The symptom-based outcome was measured based on the physician diagnosis written on the patient chart of baseline and last visit of NYHA functional class and categorized as worsened, improved and remained the same NYHA Class.

### Operational definitions

Heart failure is a complex clinical syndrome that results from any structural or functional impairment of ventricular filling or ejection of blood [2]. Hospitalization: when the patient is admitted to the medical ward or at emergency center for greater than ≥ 24 h within the study period

- Worsened: Increase patients NYHA functional class from the baseline NYHA functional class of the patient but not hospitalized.
- Improved: decreased patient NYHA functional class from baseline NYHA functional class of the patient.
- Remained the same: If the last visit NYHA functional class similar to baseline NYHA functional classes.
- Etiology: any medical condition or disease, which causes heart failure is documented in the patient medical record.
- Comorbidity: The presence of any chronic medical problem along with heart failure documented in the patient medical record.
- Adequate self-care: if the patient’s behavioral self-care scores greater or equal to 70 whereas inadequate self-care if the patient’s behavioral self-care scores less than 70 [16].
- Clinical inertia: is a lack of treatment initiation or the intensification of therapies without evidence-based clinical guidelines to achieve therapeutic goals [17].

## Result

### Socio-demographic characteristic of the patient

From 253 ambulatory chronic heart failure patients enrolled initially, six patients lost to follow-up within the first three months, and additionally five patients lost to follow-up in the next months of the study period. Totally, 11 (4.3%) patients were lost to follow-up, and 242 patients were included in the final analysis. According to this study, from the total of 242 study participants, 126 (52.1%) of patients were males. The mean standard deviation (SD) age of the patients was 53.33 ± 15.27 years. The mean (SD) body mass index (BMI) of patients was 21.53 ± 3.89 kg/m^2^ and most of the patients (81.0%) were married. With regard to educational status, more than half (55.0%) of the patients were unable to read and write and 6 (2.5%) of patients attended college and above. Of the study participants, 151 (62.4%) patients resided in rural areas. Whereas 223 (92.1%) of the patients live with their families while 15 (6.2%) of patients live alone. The mean (SD) duration since HF diagnosis was 4.09 ± 3.76 years **(**Table 1**).**

**Table 1:** Socio demographic characteristics of the study participants at JMC South West Ethiopia from November 2020 to June 2021

Variables	Category	Frequency (%)
Gender	Male	126 (52.1)
	Female	116 (47.9)
	Mean ± SD	53.3 ± 15.27
BMI (kg/m^2^)	Mean ± SD	21.53 ± 3.89
Marital status	Married	196 (81.0)
	Single	14 (5.8)
	Divorced	13 (5.4)
	Widowed	19 (7.9)
Educational status	Unable to read / write	133(55.0)
	Primary school (1–8)	76 (31.4)
	Secondary school (9–12)	27 (11.2)
	Collage and above	6 (2.5)
Home residence	Rural	151 (62.4)
	Urban	91 (37.6)
Occupation	Employee	26 (10.7)
	Farmer	123 (50.8)
	Retired	12 (5.0)
	Merchant	17 (7.0)
	Un employed	64 (26.4)
Patient living condition	Living with family	223 (92.1)
	Alone	15(6.2)
	Living with friends	4 (1.7)
Duration of heart failure diagnosis (years)	> 1	39 (16.1)
	1–5	135 (55.8)
	> 5	68 (28.1)
	Mean ± SD	4.09 ± 3.76

### Patients behavioural characteristics of study participants

From study participants, 168 (69.4%) patients had every 2-month follow-up. About 57 (23.6%) patients followed their clinical care on a monthly basis. Most of the study participants (77.3%) had inadequate behavioral self-care. The majority (53.3%) of the patients refill their prescriptions through out-of-pocket payment while 105 (43.4%) of the patients had insurance for their medicines. Of the study participants, 208 (86.0%) patients didn’t practice self-medication, and 202 (83.5%) patients never chewed chat. Of the study participants, 234 (96.7%) patients were adhering to the medication as the physician prescribed and 116 (47.9%) of patients had comorbid diseases. More than one-thirds of patients had history of hospitalization within the last year (Table 2).

**Table 2:** Behavioral characteristics of the study participants at JMC, South West Ethiopia from November 2020 to June 2021

Variables	Category	Frequency (%)
Follow up interval	Monthly	57 (23.6)
	Every 2 month	168 (69.4)
	Every 3 month	17 (7.0)
Ways of getting medication	Out of pocket payment	129 (53.3)
	For free	8 (3.3)
	Insurance	105 (43.4)
Self-medication practice	Yes	34 (14.0)
	No	208 (86.0)
Self-care practice	Adequate	187 (77.3)
	Inadequate	55 (22.7)
Chat chewing	Never	202 (83.5)
	Weekly	33 (13.6)
	Daily or almost daily	7 (2.9)
Alcohol drink	Never	235 (97.1)
	Once\ twice per month	4 (1.7)
	Weekly	2 (0.8)
	Daily or almost daily	1 (0.4)
Adhere to follow up schedule	Yes	224 (92.6)
	No	18 (7.4)
Family history of heart failure	Yes	26 (10.7)
	No	216 (89.3)
History of hospitalization within last one year	Yes	88 (36.4)
	No	154(63.6)
Presence of comorbid disease	Yes	116 (47.9)
	No	126 (52.1)

### Etiologies of HF ambulatory heart failure patients

**Fig. 1:**
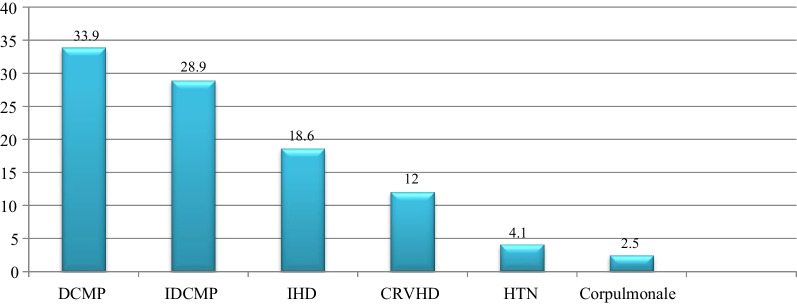
Percentage of etiology of ambulatory heart failure patients. *CRVHD* chronic rheumatic valvular heart diseases, *DCMP* dilated cardio myopathy, *IDCMP* ischemic dilated cardio myopathy, *IHD* ischemic heart disease

Among 242 heart failure patients, 82 (33.9%) of HF was secondary to dilated cardiomyopathy (DCMP), and 70 (28.9%) of HF was secondary to ischemic dilated cardiomyopathy (IDCMP). Whereas 45 (18.8%) of the etiology was ischemic heart diseases (IHD) while 29 (12.0%) were due to chronic rheumatic valvular heart disease and 10 (4.1%) patients secondary to hypertension. Cor-pulmoale was the least etiology of HF in this study (Fig. 1).

### Baseline NYHA class, blood pressure, and echo-finding study participants

The mean (SD) systolic blood pressure (SBP) of the patient was (115.98 ± 19.17 mmHg. Whereas the mean (SD) diastolic blood pressure (DBP) was 74.68 ± 13.58 mmHg. The majority of HF the patients (69.8%) were in functional NYHA class I at baseline while (22.7%) of patients were in functional NYHA class II and (7.4%) of patients were in functional NYHA class III. The mean (SD) left ventricular ejection fraction of the patient was 37.93 ± 13.59. From study participants, approximately one-third of heart failure patients complaints were easy fatigability. Whereas 26.3% of study participants had shortness of breath and 5.8% of patients compliant were peripheral edema (Table 3).

**Table 3:** Base line NYHA class, blood pressure and echo finding of ambulatory heart failure patients at JMC, South West Ethiopia from November 2020 to June 2021

Variables	Category	Frequency (%)
SBP (mmHg)	Mean ± SD	115.98 ± 19.17
DBP (mmHg)		74.68 ± 13.58
Functional NYHA class	I	169 (69.8)
	II	55 (22.7
	III	18 (7.4)
	IV	0
Common complaints of study participants	Easy fatigability	71 (31.7)
	Shortness of breath	59 (26.3)
	Cough	28 (12.5)
	Anorexia	29 (12.9)
	Orthopnea	11 (4.9)
	Peripheral edema	13 (5.8)
	Palpation	7 (3.1)
	Other	6 (2.7)
Echo finding	LVEF (%)	37.93 ± 13.59
	LAD (cm)	3.72 ± 1.10
	LVIDd (cm	5.69 ± 1.11
	LVIDs (cm)	4.71 ± 1.26

*DBP* diastolic blood pressure**,** *LAD* left atrium diameter, *LVIDd* left ventricle internal diameter end diastole, *LVIDs* left ventricle internal diameter end systole, *SBP* systolic blood pressure

### Baseline heart failure medication prescribed to ambulatory heart failure patients

The patterns of heart failure medications prescribed for study participants, beta-blockers were prescribed for 90.1% of study participants and 87.2% of study participants were taking angiotensin-converting enzyme inhibitors/angiotensin receptor blockers. Whereas mineralocorticoid receptor antagonist was the most common unprescribed class of medication from HF medications. Loop diuretics were prescribed for only 71.2% of study participants (Table 4).

**Table 4:** Base line common heart failure medications prescribed at ambulatory heart failure patients at JMC, South West Ethiopia from November 2020 to June 2021

Drug class	Category	Frequency (%)
Beta blocker	Prescribed	218 (90.1)
	Not-prescribed	24 (9.9)
ACEIs/ARB	Prescribed	211 (87.2)
	Not-prescribed	31 (12.8)
Loop diuretics	Prescribed	172 (71.1)
	Not-prescribed	70 (28.9)
MRA	Prescribed	84 (34.7)
	Not-prescribed	158 (65.3)
Digoxin	Prescribed	10 (4.1)
	Not-prescribed	132 (94.9)

*ACEI* angiotensin converting enzyme inhibitor, *ARB* angiotensin receptor blocker, *MRA* mineralocorticoid receptor antagonists

### Treatment outcome of ambulatory heart failure patients

**Fig. 2:**
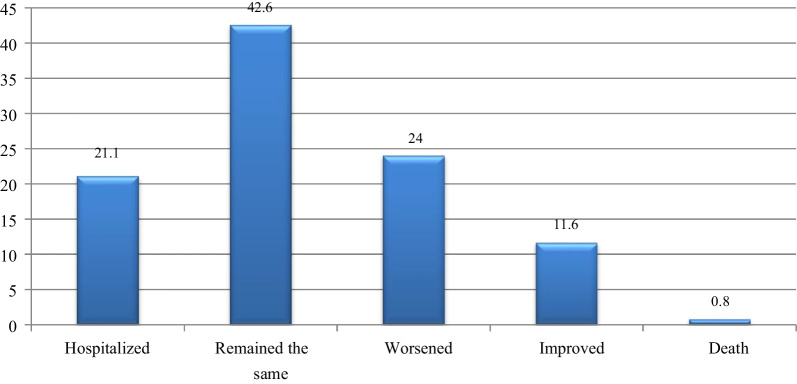
Percentage of treatment outcome of ambulatory heart failure patients at JMC, South West Ethiopia from November 2020 to June 2021

The primary outcome of this study was the hospitalization of ambulatory heart failure patients. Among 242 chronic ambulatory heart failure patients, 51 (21.1%) of patients were hospitalized within the study period. Of the study participants, 103 (42.6%) patients remained in the same NYHA functional class baseline. Whereas 58 (24.0%) and 28 (11.6% of patients worsened and improved respectively compared to baseline NYHA functional class and 2 (0.8) patients died (Fig. 2).

### Factors associated with hospitalization of ambulatory heart failure patients

In this study, history of hospitalization, self-medication practice, absence of beta-blocker at first the visit, and MRA dose < 25% of the targeted dose at baseline were a risk factors for hospitalization of heart failure patients in univariate logistic regression. And multivariate logistic regression, clinical inertia, baseline NYHA class II and out-of-pocket payment were independent variables significantly associated with hospitalization. From study participants, patients who had clinical inertia within the study period had an increased risk of being hospitalized by three times compared to those patients who didn’t have clinical inertia [AOR 2.820; 95% CI (1.301, 6.110); *p* = 0.009]. Patients who got their medication out-of-pocket had an increment of hospitalization approximately by three times compared to those patients who got their medication through insurance coverage [AOR 2.790; 95% CI (1.261, 6.172), *p* = 0.011], and patients with NYHA class II at baseline the risk of hospitalization increased by three times compared to a patient with NYHA class I [AOR 2.534; 95% CI (1.170, 5.488), *p* = 0.018] (Table 5).

**Table 5:** Univariate and multivariate logistic regression analysis for hospitalization of ambulatory chronic heart failure patients

Variables	Category	Hospitalization		COR	*p* value	AOR	*p* value
		No	Yes				
Clinical inertia	No	98	14	1		1	
	Yes	93	37	2.785 (1.415, 5.482)	0.003	2.820 (1.301, 6.110)	0.009*
Occupational status	Gov’t employed			1		1	
	Farmer			0.430 (0.131–0.419)	0.166	0.023 (0.002–0.466)	0.012
	Retired			0.487 (0.239–0.994)	0.048	0.178 (0.042–0.742)	0.720
	Merchant			0.215 (0.026–1.786)	0.154		0.998
	Unemployed			1.291 (0.417–3.999)	0.656	0.232 (0.026–2.064)	0.190
Base line NYHA class	Class I	142	27	1		1	
	Class II	36	19	2.776 (1.390, 5.542)	0.004	2.534 (1.170, 5.488)	0.018*
	Class III	13	5	2.023 (0.666, 6.141)	0.214	1.136 (0.267, 4.836)	0.863
Comorbid	No			1		1	
	Yes			1.056 (5.694–19.604)	0.243	0.494 (0.320- 1.733)	0.494
Self-medication	Yes	21	13	2.769 (1.275, 6.017)	0.010	2.362 (0.947, 5.888)	0.065
	No	170	38	1		1	
Hospitalization history	No	126	28	1		1	
	Yes	65	23	2.163 (1.156–4.050)	0.015	1.529 (0.733, 3.189)	0.258
Base line spiranoloaclone	Yes	71	15	1		1	
	No	120	36	5.037 (0.739, 34.341)	0.099	6.047 (0.768, 47.584)	0.087
Ways of getting medication	Insurance	93	12	1		1	
	Out of pocket	92	37	3.117 (1.117, 6.353)	0.002	2.79 0(1.261, 6.172)	0.011*
	For free	6	2	2.583 (0.467, 14.247)	0.277	2.176 (0.347, 13.641)	0.406
Beta blocker prescribed at base line	Yes	175	41	1		1	
	No	16	10	2.668 (1.129, 6.305)	0.025	2.356 (0.900, 6.166)	0.081

Note: *Statistically significant

## Discussion

Heart failure has been one of the major causes of hospitalization and mortality across the world. It is one of the challenging public health problems of the twenty-first century and is associated with poor outcomes such as death and hospitalization. Giving more attention to ambulatory HF patients will help reducing and preventing HF-related morbidity and mortality [18]. This study aimed to assess treatment outcomes and associated factors to hospitalization of ambulatory HF patients at JMC. As the result of this study shown, hospitalization, clinical inertial and worsening of ambulatory HF patients frequently occurred. More than half HF patients had clinical inertia within the study period. Clinical inertia, out-of-pocket payment and NYHA class II were independent predictors of hospitalization of ambulatory HF patients.

According to this study, among 242 ambulatory HF patients, 51 (21.1%) were hospitalized within the six months of the study period. Fifty-eight (24.0%) of the patient had worsened; 28 (11.6%) of patients had improved and 103 (42.6%) had remained in the same NYHA class compared to the baseline patient NYHA class. The results of this study showed, worsened patients higher than that conducted in the USA and Spain, the prevalence of worsened HF patients were occurred at 2.9 and 16.9% respectively [19, 20]. The discrepancy might be that both countries are developed and they may have advanced and experienced HF management setting which could be the reason for the occurrence of worsening HF patients decreased in the comparative studies. The other possible reason might be patient behavioral self-care practice of ambulatory HF patients in this study was inadequate which could be increased the worsening of ambulatory HF patients in this study. With regard to hospitalization, the finding of this study was lower than a retrospective cohort study conducted in USA, hospitalization occurred 52.7% [13]. The variation might be the large sample size (5139) HF, long duration of the study (38 months), and most patients were aged (mean 64.3) years and obese (body mass index 28.5 kg/m^2^) due to these conditions’ hospitalization might be increased in the comparative study. Similarly, the finding of this study was lower than the study conducted in Canada 38% [15], Italy 44.4% [21], England 55% [22], and India 35.5% of HF patients [23]. The discrepancy might be due to the large proportion of sample size, and most patients were aged which could be increased by hospitalized in the comparative studies. The other possible justification for the discrepancy might be there were re-hospitalized patients as well as long duration of the study period may increase the rate of hospitalized ambulatory HF patients in comparative studies.

The rate of hospitalization in this study is almost in line with a study conducted in Israel hospitalization was 21.6% [12]. The finding of this study was higher than the study conducted in different countries rate hospitalization of chronic HF patients was in Canada 18% [24], in Spain 9.0% [14], and in France 18% [25]. The discrepancy might be, those countries are more developed, they may have a better and advanced HF management setting compared to this study setting. But half of ambulatory HF patients had clinical inertia or lack of advanced management. Inadequate self-care behavior practice might be increased being hospitalized in this study compared to comparative studies. Similarly, the rate of hospitalization in this study was also higher than a hospital-based prospective study conducted in Nigeria readmission rate at 30 and 180 days were 1.53% and 12.5% from 262 ambulatory HF patients [4]. The discrepancy might be relatively long duration of the study period will increase the rate of being hospitalized in this study. The other possible reason might be Nigeria relatively developing country as a result better HF management setting might be there which important to prevent hospitalization ambulatory HF patients in Nigeria compared to this study setting.

In this study, clinical inertia, NYHA functional class II and out-of-pocket payment were independently associated with hospitalization among ambulatory HF patients. Whereas a retrospective cohort study in Catalonia (North-East Spain) chronic kidney diseases (CKD), chronic obstructive pulmonary diseases (COPD) and diabetic mellitus were identified as predictors of hospitalization of in ambulatory HF patients [14]. The discrepancy might be small sample size as well as patients characteristics (CKD, COPD and diabetic mellitus) comorbid patients were low in number among this study participants.

In this study clinical inertia was detected as a significantly associated for hospitalization of ambulatory HF patients at [AOR = 2.820; with 95% CI (1.301, 6.110) *p* = 0.009], patients with clinical inertia three times increased the risk of hospitalization compared to HF patients without clinical inertia. More than 50% of study participants had clinical inertia at a certain point within the study period. Because of lack of intensification or initiation of heart failure medications increased the risk of hospitalization of ambulatory HF patients. American College of Cardiology (ACC), AHA and the European Society of Cardiology (ESC) all issue clear HF guidelines to support evidence-based care to HF patients decreased heart failure burden both morbidity and mortality of heart failure. This study was supported by a similar study conducted in Tanzania showed that absence of ACEI/ARB was risk factor associated for hospitalization HF patients [26].

According to this study, patients got their medication through out-of-pocket payment was a significant predictable factor for hospitalization [AOR = 2.790, with 95% CI (1.261, 6.172); *p* = 0.011] approximately three times more likely for hospitalization compared to patients got their medication through insurance coverage. This might be due to affordability issues of the medications or under use of the medication could be increased hospitalization of HF patients. Most of the patients in study were farmers and reside in rural areas, they may have a problem of medication-related cost issue that causes underuse or un-able to afford the medication which increased risk of being hospitalized HF patients. The finding of this study supported with the study conducted in United States, medication under use because of cost was significantly more likely to report being hospitalized with higher the adjusted predicted probability of being hospitalized [27]. The other variable significantly associated with hospitalization in this study was base line NYHA class II. NYHA functional class II was detected as independently associated with hospitalization of ambulatory HF patients at [AOR = 2.534; 95% CI (1.170, 5.488), *p* = 0.018] in which the risk of hospitalization increased by three times compared to base line NYHA class I HF patients. Scientifically, HF patients with higher NYHA class patients had or increased sign and symptoms of HF which more likely to being hospitalized. This study was supported by a study conducted in USA; NYHA class II was detected as a predictor of associated hospitalization among patients with preserved left ventricular function [28]. Study conducted in the same country, higher NYHA class (III/IV) of patient were among predictors of hospitalization of HF patients [13]. The discrepancy might be, study conducted in large sample size, and large numbers of patients were in NYHA class III and IV in the comparative study.

## Conclusion

As the results of this study showed hospitalization, clinical inertial, and worsening of ambulatory HF patients frequently occurred within the study period at Jimma Medical Center. This study showed that hospitalization was high among ambulatory heart failure patients. Ten percent heart failure patients were worsened. Generally, more than one-third of ambulatory heart failure patients had either hospitalized or worsening symptom in six months of follow up compared to base line of patient status. More than half of patients had clinical inertia. And also, this study showed most ambulatory HF patients had inadequate self-care. Clinical inertia, out of pocket payment and NYHA class II were independently predictors of hospitalization in ambulatory heart failure patients.

### Recommendation

Based on the findings of this study, we would like to forward our suggestions to concerned bodies. Health professionals, especially physicians working at ambulatory HF patients: better to adhere to standard guideline of HF management to minimize clinical inertia, and give regular education on self-care behavior practice to prevent frequent hospitalization and burden of ambulatory heart failure patients. Further researches: shall focusing on ambulatory HF patients in large scale level of studies to further quantify clinical inertia and outcomes of HF patients.

### Strength and limitation of the study

The strength of this study employed a prospective study design, conducted on active follow-up patients with regular follow-until study period ended. This study assessed patients self-care behavior practice, clinical inertia and identifies factors associated with hospitalization in ambulatory heart failure patients. Small sample size, a single study setting and short study period were some limitations of this study. Additionally, even if adherence of HF patients assessed combined with self-care behavioral practice tool, independently adherence measurement tool was not used.

## Data Availability

Data used and analyzed during the current study are available from corresponding author on reasonable request.

## Abbreviations

ACEI: Angiotensin converting enzyme inhibitor
BB: Beta blocker
BMI: Body mass index
CHF: Chronic heart failure
CKD: Chronic kidney disease
COPD: Chronic obstructive pulmonary diseases
HF: Heart failure
JMC: Jimma Medical Center
MRA: Mineralocorticoid receptor antagonists
NYHA: New York Heart Association
SSA: Sub Sahara Africa
